# Effect of antiretroviral therapy use and adherence on the risk of hyperlipidemia among HIV-infected patients, in the highly active antiretroviral therapy era

**DOI:** 10.18632/oncotarget.22465

**Published:** 2017-11-15

**Authors:** Fuu-Jen Tsai, Chi-Fung Cheng, Chih-Ho Lai, Yang-Chang Wu, Mao-Wang Ho, Jen-Hsien Wang, Ni Tien, Xiang Liu, Hsinyi Tsang, Ting-Hsu Lin, Chiu-Chu Liao, Shao-Mei Huang, Ju-Pi Li, Jung-Chun Lin, Chih-Chien Lin, Jin-Hua Chen, Wen-Miin Liang, Ying-Ju Lin

**Affiliations:** ^1^ School of Chinese Medicine, China Medical University, Taichung, Taiwan; ^2^ Genetic Center, Department of Medical Research, China Medical University Hospital, Taichung, Taiwan; ^3^ Asia University, Taichung, Taiwan; ^4^ Graduate Institute of Biostatistics, School of Public Health, China Medical University, Taichung, Taiwan; ^5^ Department of Microbiology and Immunology, Chang Gung University, Taoyuan, Taiwan; ^6^ Section of Infectious Diseases, Department of Internal Medicine, China Medical University Hospital, Taichung, Taiwan; ^7^ Department of Medical Laboratory Science and Biotechnology, China Medical University, Taichung, Taiwan; ^8^ National Institute of Allergy and Infectious Diseases, National Institutes of Health, Bethesda, Maryland, USA; ^9^ Rheumatism Research Center, China Medical University Hospital, Taichung, Taiwan; ^10^ School of Medical Laboratory Science and Biotechnology, College of Medical Science and Technology, Taipei Medical University, Taipei, Taiwan; ^11^ Department of Cosmetic Science, Providence University, Taichung, Taiwan; ^12^ Biostatistics Center, College of Management, Taipei Medical University, Taipei, Taiwan; ^13^ School of Health Care Administration, College of Management, Taipei Medical University, Taipei, Taiwan

**Keywords:** HIV, antiretroviral therapy, hyperlipidemia, nucleoside reverse-transcriptase inhibitor, protease inhibitor

## Abstract

HIV-infected patients exposed to antiretroviral therapy (ART) have an increased risk for hyperlipidemia and cardiovascular disease. We performed a longitudinal, comprehensive, and population-based study to investigate the cumulative effect of different types of ART regimens on hyperlipidemia risk in the Taiwanese HIV/ART cohort. A total of 13,370 HIV-infected patients (2,674 hyperlipidemia and 10,696 non-hyperlipidemia patients) were recruited after matching for age, gender, and the first diagnosis date of HIV infection by using the National Health Insurance Research Database in Taiwan. Hyperlipidemia risk associated with cumulative ART use, ART adherence, and their combination was assessed. The matched hyperlipidemia group had a larger number of patients using ART and a higher incidence of comorbidities, specifically, respiratory disease and diabetes. Patients with high ART dosage and dose-dependent manner adherence, respectively, demonstrated an increased risk of hyperlipidemia. For single ART regimens, patients receiving nucleoside reverse-transcriptase inhibitors (NRTI/NRTI)- containing regimen had the highest hyperlipidemia risk, followed by protease inhibitor (PI)- containing and non-NRTI- containing regimens. For combination ART regimens, patients receiving a NRTI/NRTI + PI regimen had the highest hyperlipidemia risk. An increased cumulative drug dose was observed in patients who received the PI, NRTI/NRTI, NRTI, and NNRTI regimens in the hyperlipidemia group, when compared to the non-hyperlipidemia group. In conclusion, ART cumulative use, adherence, and regimen may affect hyperlipidemia risk among HIV-infected patients in a dose-dependent manner.

## INTRODUCTION

According to reports from the WHO (World Health Organization) and the UNAIDS (The Joint United Nations Programme on HIV/AIDS), the estimated number of people living with HIV/ AIDS (PLWHA) in 2015 was 36.9 million [1]. Of these, 17 million people receive antiretroviral therapy (ART). With the aid of ART regimens, HIV replication has been effectively suppressed, and subsequent AIDS-related mortality and morbidity have reduced in these patients [2]. The use of ART has led to decreased risks of drug resistance, HIV transmission and AIDS disease progression as well as improved overall health, quality of life, and survival [3, 4].

However, adverse effects have been reported for all antiretroviral (ARV) drugs; this is one of the most common reasons for discontinuing or switching ART [5, 6]. Since ART is now recommended for all patients, regardless of CD4 T lymphocyte (CD4) cell count, awareness regarding the adverse effects of its long-term use is paramount. Some of the long-term effects include bone or renal toxicity, hyperlipidemia, diabetes mellitus, or accelerated cardiovascular disease [7–11].

Hyperlipidemia is characterized by abnormal levels of any, some, or all the lipids or lipoproteins in the blood [12]. It often occurs in HIV-infected patients who receive ART. The blood lipid profiles of such patients change within three months after receiving ART, and reach a plateau after six to nine months [13]. HIV/ART hyperlipidemia includes high total cholesterol (TC), hypertriglyceridemia (high TG), increased plasma low-density lipoprotein cholesterol (LDL-C), increased very-low-density lipoprotein (VLDL), increased levels of apolipoprotein (apoB), and variable levels of plasma high-density lipoprotein cholesterol (HDL-C) [14–16]. It has been associated with an increased risk for cardiovascular disease among these patients [14, 15, 17–20]. Studies have shown that, in HIV/ART patients, the prevalence of hyperlipidemia and the risk for cardiovascular disease range from 20% to 80% [21]. Blood lipid profile alterations were first described in patients receiving protease inhibitors (PI); however, these have also been reported in patients receiving nucleoside reverse-transcriptase inhibitors (NRTI) and non-nucleoside reverse-transcriptase inhibitors (NNRTI) [14, 22].

Since a higher prevalence of hyperlipidemia and cardiovascular disease risk was observed in HIV/ART patients, we investigated the cumulative effect of ART and the adherence of ART regimens on hyperlipidemia risk among HIV/ART patients. We used a longitudinal, comprehensive, and population-based database to investigate demographic characteristics, cumulative ART use, ART adherence, and the effects of ART on the occurrence of hyperlipidemia in the HIV infected patient cohort (1998–2012) in Taiwan. In addition, the effects of single and combination ART regimens on hyperlipidemia risk were investigated.

## RESULTS

### Characteristics of the study patients

**Figure 1:**
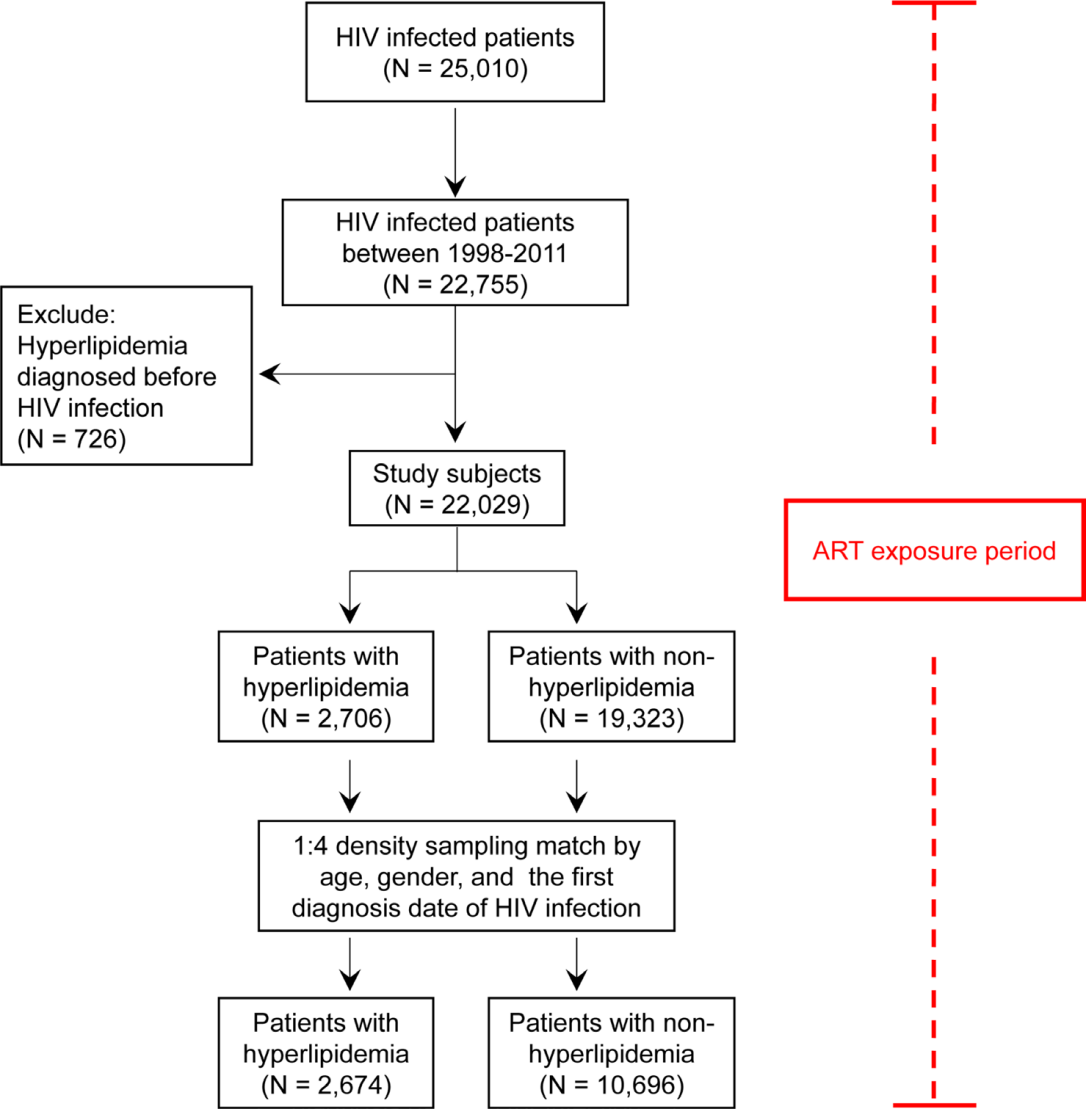
Flow recruitment diagram Chart showing the protocol for enrollment of study subjects.

Of the 25,010 HIV-infected patients, the study population consisted of 2,706 hyperlipidemia cases and 19,323 non-hyperlipidemia controls (Figure 1). The demographic characteristics of hyperlipidemia cases vs. non-hyperlipidemia controls are shown in the left side of Table 1. Differences in age, gender, follow-up years, ART usage, and comorbidities (cardio-cerebrovascular disease, respiratory diseases, diabetes, renal diseases, and liver diseases) were observed between the two groups. Comorbidities were defined as conditions present in the patients prior to their subsequent HIV diagnosis. The hyperlipidemia cases were characterized by older age, longer follow-up years, an increased incidence in males subjects, for ART use, and comorbidities (cardio-cerebrovascular disease, respiratory diseases, and diabetes), respectively (*p* < 0.001).

**Table 1:** Demographic characteristics of HIV-infected patients with and without hyperlipidemia (total subjects and density-sampling matched subjects)

Characteristics	Total subjects		*p*-value	Density sampling matched subjects		*p*-value
	Hyperlipidemia	Non-hyperlipidemia		Hyperlipidemia	Non-hyperlipidemia	
	*N* = 2,706	*N* = 19,323		*N* = 2,674	*N* = 10,696	
	*N* (%)	*N* (%)		*N* (%)	*N* (%)	
**Gender**			***<0.001***			1
Male	2358 (87.14%)	15257 (78.96%)		2345 (87.7%)	9380 (87.7%)	
Female	348 (12.86%)	4066 (21.04%)		329 (12.3%)	1316 (12.3%)	
**Age**			***<0.001***			0.736
<50	2426 (89.65%)	18420 (95.33%)		2420 (90.5%)	9657 (90.29%)	
≥50	280 (10.35%)	903 (4.67%)		254 (9.5%)	1039 (9.71%)	
**Follow-up years**	8.40 ± 3.79		***<0.001***	4.35 ± 3.19	4.35 ± 3.19	1
**ART usage**			***<0.001***			***<0.001***
Non-ART use	668 (24.69%)	10591 (54.81%)		709 (26.51%)	6062 (56.68%)	
ART use	2038 (75.31%)	8732 (45.19%)		1965 (73.49%)	4634 (43.32%)	
**Comorbidities**						
Cardio-cerebrovascular diseases	87 (3.22%)	414 (2.14%)	***<0.001***	77 (2.88%)	247 (2.31%)	0.086
Respiratory diseases	190 (7.02%)	1045 (5.41%)	***<0.001***	183 (6.84%)	511 (4.78%)	***<0.001***
Rheumatological diseases	19 (0.7%)	89 (0.46%)	0.092	18 (0.67%)	60 (0.56%)	0.496
Digestive diseases	237 (8.76%)	1573 (8.14%)	0.273	227 (8.49%)	859 (8.03%)	0.438
Diabetes	103 (3.81%)	269 (1.40%)	***<0.001***	96 (3.59%)	204 (1.91%)	***<0.001***
Renal diseases	22 (0.81%)	99 (0.51%)	0.047	20 (0.75%)	87 (0.81%)	0.734
Liver diseases	215 (7.95)	1760 (9.11%)	0.047	212 (7.93%)	869 (8.12%)	0.739
Cancer	38 (1.4%)	208 (1.08%)	0.129	37 (1.38%)	126 (1.18%)	0.386
**Anti-hyperlipidemia drug**						
Statin	604 (22.32%)	752 (3.89%)	***<0.001***	574 (21.47%)	387 (3.62%)	***<0.001***
Fibrate	509 (18.82%)	529 (2.74%)	***<0.001***	488 (18.25%)	283 (2.65%)	***<0.001***

*N*, number; ART, antiretroviral therapies.

*p*-values were obtained by the chi-square test; *p*-values for the follow-up years were obtained by the un-paired Student *t*-test.

Significant *p-*values (*p* < 0.05) are highlighted in bold italic font.

Comorbidities present in the patients prior to their subsequent HIV diagnosis were defined as follows: cardio-cerebrovascular disease (ICD-9-CM: 410, 412, 428, 441, 443.9, 430–438, 785.4, V43.4, and 38.48 (P)), respiratory diseases (ICD-9-CM: 490–496, 500–505, and 506.4), rheumatic diseases (ICD-9-CM: 710.0, 710.1, 710.4, 714.0–714.2, 714.81, and 725), digestive diseases (ICD-9-CM: 531–534), diabetes (ICD-9-CM: 250.0–250.3, and 250.7), renal disease (ICD-9-CM: 582, 583–583.7, 585, 586, and 588), liver diseases (ICD-9-CM: 571.2, 571.4–571.6, 070.4, 070.5, and 070.7), and cancer (ICD-9-CM: 140-172, 174–195.8, and 200–208).

The incidence-density sampling match method was used to match the hyperlipidemia and non-hyperlipidemia groups. After matching these two groups for age, gender, and the first diagnosis date of HIV infection, 2,674 hyperlipidemia and 10,696 non-hyperlipidemia patients were included in this analysis (Figure 1 and Table 1 right side). There were no differences in age, gender, and follow-up years between these two groups. However, there were differences in the frequency distributions of ART usage and in respiratory disease and diabetes comorbidities (*p* < 0.001). In the hyperlipidemia group, 73.48% used ART, while only 43.33% were reported to use ART in the non-hyperlipidemia group. Furthermore, in the hyperlipidemia group, 6.84% of patients had respiratory diseases and 3.59% had diabetes, compared to 4.78% and 1.91%, respectively, in the non-hyperlipidemia group. These results suggest that the matched hyperlipidemia group was characterized by a higher number of patients using ART and an increased incidence of respiratory diseases and diabetes.

### Hyperlipidemia risk in HIV-infected patients according to ART cumulative dose, adherence, and their combination

As shown in Figure 1 and Table 1, Taiwanese HIV-infected patients with hyperlipidemia were characterized by higher ART use and a higher number of cases with comorbidities, specifically respiratory diseases and diabetes, even after matching for age, gender, and the first diagnosis date of HIV infection. In order to investigate the effect of ART usage on hyperlipidemia risk among Taiwanese HIV-infected patients, cumulative ART dose, adherence, and their combination were examined (Table 2). The univariate logistic regression model revealed cumulative dose, adherence, and cumulative dose* adherence of ART to be associated with hyperlipidemia risk in a dose-dependent manner (*p* < 0.0001; Table 2).

**Table 2:** Hyperlipidemia risk in HIV-infected patients according to the cumulative ART dose, adherence, and their combination

Cumulative dose and adherence	Hyperlipidemia	Non-hyperlipidemia	Univariate			Multiple		
	*N* = 2,674	*N* = 10,696	OR	95% CI	*p*-value	OR	95% CI	*p*-value
	*N* (%)	*N* (%)						
**ART cumulative dose**								
Non-ART use	709 (26.5%)	6062 (56.7%)	1	ND	ND	1	ND	ND
ART cumDDDs < 1000	513 (19.2%)	1541 (14.4%)	3.65	(3.15–4.22)	***<0.0001***	3.74	(3.22–4.33)	***<0.0001***
ART cumDDDs ≥ 1000	1452 (54.3%)	3093 (28.9%)	6.9	(6.07–7.84)	***<0.0001***	7.08	(6.22–8.06)	***<0.0001***
**ART adherence**								
ART adherence < 0.5	927 (34.7%)	7047 (65.9%)	1	ND	ND	1	ND	ND
0.5 ≤ ART adherence < 0.8	193 (7.2%)	667 (6.2%)	2.87	(2.38–3.45)	***<0.0001***	2.91	(2.41–3.50)	***<0.0001***
ART adherence ≥ 0.8	1554 (58.1%)	2982 (27.9%)	5.19	(4.66–5.77)	***<0.0001***	5.27	(4.72–5.87)	***<0.0001***
ART cumulative dose* adherence								
Non-ART use	709 (26.5%)	6062 (56.7%)	1	ND	ND	1	ND	ND
ART cumDDDs < 1000, adherence < 0.8	205 (7.7%)	967 (9.0%)	2.61	(2.17–3.14)	***<0.0001***	2.68	(2.22–3.26)	***<0.0001***
ART cumDDDs ≥ 1000, adherence < 0.8	206 (7.7%)	686 (6.4%)	4.28	(3.50–5.24)	***<0.0001***	4.43	(3.62–5.43)	***<0.0001***
ART cumDDDs < 1000, adherence ≥ 0.8	308 (11.5%)	574 (5.4%)	5.83	(4.75–7.16)	***<0.0001***	5.97	(4.86–7.35)	***<0.0001***
ART cumDDDs ≥ 1000, adherence ≥ 0.8	1246 (46.6%)	2407 (22.5%)	6.93	(6.08–7.90)	***<0.0001***	7.11	(6.23–8.12)	***<0.0001***

*N*, number; ART, antiretroviral therapy; OR, odds ratio; CI, confidence interval; ND, not determined; cumDDDs, cumulative defined daily doses.

The definition of ART cumulative dose: the total dose of antiretroviral drugs given to a patient from the start of ART to the study end.

The definition of ART adherence: (total number of prescribed days of a patient from the start of ART to the study end)/(total number of days of observation of a patient from the start of ART to the study end).

Adjusted for age, gender, Charlson’s comorbidity index; model selected by stepwise logistic regression.

Significant *p*-values (*p < 0.0001*) are highlighted in bold italic font.

Patients with cumulative ART defined daily doses (DDDs) ≥ 1000 had the highest hyperlipidemia risk, with an odds ratio (OR) of 6.90 (95% confidence interval [CI] of 6.07–7.84), while patients with cumulative ART DDDs < 1000 had a lesser though still elevated hyperlipidemia risk, with an OR of 3.65 (95% CI: 3.15–4.22), when compared to HIV-infected patients who did not use ART. Regarding ART adherence, patients with ART adherence ≥ 0.8 had the highest hyperlipidemia risk, with an OR of 5.19 (95% CI: 4.66–5.77); patients with 0.5 ≤ ART adherence <0.8 had a higher hyperlipidemia risk, with an OR of 2.87 (95% CI: 2.38–3.45), when compared with those HIV infected patients whose ART adherence < 0.5. Regarding cumulative ART dose* adherence, patients with cumulative ART DDDs ≥ 1000 and ART adherence ≥ 0.8 had the highest hyperlipidemia risk, with an OR of 6.93 (95% CI: 6.08–7.90) when compared to HIV-infected patients who did not use ART. Patients with cumulative ART DDDs < 1000 and ART adherence ≥ 0.8 had had the next highest risk of hyperlipidemia (OR: 5.83, 95% CI: 4.75–7.16) and those with cumulative ART DDDs ≥ 1000 and ART adherence < 0.8 (OR of 4.28, 95% CI: 3.50–5.24). Patients with cumulative ART DDDs < 1000 and ART adherence < 0.8 had a higher hyperlipidemia risk, with an OR of 2.61 (95% CI: 2.17–3.14), when compared to HIV-infected patients who did not use ART.

Even after adjusting for age, gender and Charlson’s comorbidity in the multivariable logistic regression model, cumulative ART dose, adherence to ART, and cumulative dose* adherence were associated with increased hyperlipidemia risk in a dose-dependent manner (Table 2; *p* < 0.0001). For cumulative ART dose, patients with cumulative ART DDDs ≥ 1000 had the highest hyperlipidemia risk, with an OR of 7.08 (95% CI: 6.22–8.06), while patients with cumulative ART DDDs < 1000 had a higher hyperlipidemia risk, with an OR of 3.74 (95% CI: 3.22–4.33), when compared to HIV-infected patients who did not use ART. For ART adherence, patients with ART adherence ≥ 0.8 had the highest hyperlipidemia risk, with an OR of 5.27 (95% CI: 4.72–5.87), while patients with 0.5 ≤ ART adherence < 0.8 had a higher hyperlipidemia risk, with an OR of 2.91 (95% CI: 2.41–3.50) when compared with HIV-infected patients whose ART adherence was < 0.5.

For cumulative ART dose* adherence in the multivariate model, patients with cumulative ART DDDs ≥ 1000 and an ART adherence ≥ 0.8 again had the highest hyperlipidemia risk, with an OR of 7.11 (95% CI: 6.23–8.12) followed by patients with cumulative ART DDDs < 1000 and ART adherence ≥ 0.8 (OR: 5.97, 95% CI: 4.86–7.35), patients with cumulative ART DDDs ≥ 1000 and ART adherence < 0.8 (OR:4.43, 95% CI: 3.62–5.43) and patients with cumulative ART DDDs < 1000 and ART adherence < 0.8 (OR: 2.68, 95% CI: 2.22–3.26) when compared with HIV-infected patients who did not use ART. These results suggest that patients were at a higher risk of hyperlipidemia when they were administered a higher ART dosage and had greater dose-dependent adherence, using univariate and multivariate regression analyses.

### Hyperlipidemia risk in HIV-infected patients according to ART regimen

Different types of ART regimens revealed similar trends for hyperlipidemia risk by using univariate and multivariable logistic regression models (Tables 3 and 4). Single ART regimens were still associated with hyperlipidemia risk even after adjusting with age, gender, and Charlson’s comorbidity in the multivariable logistic regression model (Table 3). Patients receiving a NRTI/NRTI-containing regimen had the highest hyperlipidemia risk, with an OR of 3.25 (95% CI: 2.96–3.57). Patients receiving a PI- containing regimen had a higher hyperlipidemia risk, with an OR of 2.81 (95% CI: 2.57–3.08), while patients receiving a NNRTI- containing regimen had a higher hyperlipidemia risk, with an OR of 1.94 (95% CI: 1.77–2.12). Patients receiving a NRTI- containing regimen had a higher hyperlipidemia risk, with an OR of 1.92 (95% CI: 1.75–2.12). Patients receiving other ART regimens (raltegravir and enfuvirtide) had a higher hyperlipidemia risk, with an OR of 1.35 (95% CI: 0.14–12.96).

**Table 3:** Hyperlipidemia risk in HIV-infected patients according to single type of art regimen

ART regimen	Hyperlipidemia *N* = 2,674	Non-hyperlipidemia *N* = 10,696	Univariate			Multiple		
	*N* (%)	*N* (%)	OR	95% CI	*p*-value	OR	95% CI	*p*-value
Non-ART use	709 (26.51%)	6,062 (56.68%)	0.28	(0.25–0.30)	***<0.0001***	0.23	(0.21–0.26)	***<0.0001***
Single type of ART regimen								
NNRTI	1,169 (43.72%)	3,126 (29.23%)	1.88	(1.72–2.05)	***<0.0001***	1.94	(1.77–2.12)	***<0.0001***
NRTI	849 (31.75%)	2,114 (19.76%)	1.89	(1.72–2.08)	***<0.0001***	1.92	(1.75–2.12)	***<0.0001***
NRTI/NRTI	1,710 (63.95%)	4,026 (37.64%)	2.94	(2.69–3.21)	***<0.0001***	3.25	(2.96–3.57)	***<0.0001***
PI	1,377 (51.5%)	3,059 (28.6%)	2.65	(2.43–2.89)	***<0.0001***	2.81	(2.57–3.08)	***<0.0001***
Other ART	7 (0.26%)	15 (0.14%)	1.33	(0.14–12.83)	0.803	1.35	(0.14–12.96)	0.796

*N*, number; ART, antiretroviral therapy; OR, odds ratio; CI, confidence interval; NNRTI, non-nucleoside reverse transcriptase inhibitors; NRTI, nucleoside/nucleotide reverse transcriptase inhibitors; PI, protease inhibitors.

Adjusted for age, gender, Charlson’s comorbidity.

Significant *p*-values (*p* < 0.05) are highlighted in bold italic font.

NNRTI includes efavirenz, etravirine, and nevirapine; NRTI includes didanosine, stavudine, abacavir, lamivudine, zidovudine, zalcitabine, tenofovir disoproxil, and emtricitabine; PI includes lopinavir and ritonavir, atazanavir, ritonavir, nelfinavir, indinavir, saquinavir, darunavir, and tipranavir; Other ART includes raltegravir and enfuvirtide.

**Table 4:** Hyperlipidemia risk in HIV-infected patients according to combination of 2 ART regimens

ART regimen	Hyperlipidemia	Non-hyperlipidemia	Univariate			Multiple		
	*N* = 2,674	*N* = 10,696						
	*N* (%)	*N* (%)	OR	95% CI	*p*-value	OR	95% CI	*p*-value
**Non-ART use**	709 (26.51%)	6,062 (56.68%)	0.24	(0.22–0.26)	***<0.0001***	0.22	(0.21–0.24)	***<0.0001***
**2-combinations of ART regimen**								
**NNRTI + NNRTI**	9 (0.34%)	28 (0.26%)	1.39	(0.65–2.96)	0.3991	1.35	(0.63–2.90)	0.4386
**NNRTI + NRTI**	741 (27.71%)	2,110 (19.73%)	1.49	(1.35–1.64)	***<0.0001***	1.49	(1.35–1.64)	***<0.0001***
**NNRTI + NRTI/NRTI**	931 (34.82%)	2,768 (25.88%)	1.52	(1.39–1.67)	***<0.0001***	1.53	(1.40–1.68)	***<0.0001***
**NNRTI + PI**	148 (5.53%)	507 (4.74%)	1.18	(0.97–1.42)	0.0911	1.17	(0.97–1.42)	0.1018
**NRTI + NRTI**	777 (29.06%)	1,955 (18.28%)	1.83	(1.66–2.02)	***<0.0001***	1.86	(1.68–2.05)	***<0.0001***
**NRTI + NRTI/NRTI**	172 (6.43%)	644 (6.02%)	1.07	(0.90–1.28)	0.4327	1.05	(0.89–1.26)	0.5577
**NRTI + PI**	936 (35%)	2,461 (23.01%)	1.67	(1.53–1.82)	***<0.0001***	1.69	(1.55–1.85)	***<0.0001***
**NRTI/NRTI + NRTI/NRTI**	20 (0.75%)	64 (0.60%)	1.32	(0.81–2.13)	0.2654	1.33	(0.82–2.16)	0.2494
**NRTI/NRTI + PI**	1,283 (47.98%)	2,990 (27.95%)	2.25	(2.06–2.45)	***<0.0001***	2.31	(2.12–2.52)	***<0.0001***
**PI + PI**	308 (11.52%)	800 (7.48%)	1.61	(1.40–1.85)	***<0.0001***	1.62	(1.41–1.86)	***<0.0001***

*N*, number; ART, antiretroviral therapy; OR, odds ratio; CI, confidence interval; NNRTI, non-nucleoside reverse transcriptase inhibitors; NRTI, nucleoside/nucleotide reverse transcriptase inhibitors; PI, protease inhibitors.

Adjusted for age, gender, Charlson’s comorbidity.

Significant *p*-values (*p* < 0.05) are highlighted in bold italic font.

NNRTI includes efavirenz, etravirine, and nevirapine; NRTI includes didanosine, stavudine, abacavir, lamivudine, zidovudine, zalcitabine, tenofovir disoproxil, and emtricitabine; PI includes lopinavir and ritonavir, atazanavir, ritonavir, nelfinavir, indinavir, saquinavir, darunavir, and tipranavir; Other ART includes raltegravir and enfuvirtide.

After adjusting for age, gender and Charlson’s comorbidity, in the multivariable logistic regression model, the two-combination ART regimens were associated with hyperlipidemia risk (Table 4). Patients receiving a NRTI/NRTI + PI regimen had the highest hyperlipidemia risk, with an OR of 2.31 (95% CI: 2.12–2.52). This was followed by patients receiving a NRTI + NRTI regimen (OR: 1.86, 95% CI: 1.68–2.05), patients receiving a NRTI + PI regimen (OR: 1.69, 95% CI: 1.55–1.85), patients receiving a PI + PI regimen (OR: 1.62, 95% CI: 1.41–1.86), patients receiving a NNRTI + NRTI/NRTI regimen (OR: 1.53, 95% CI: 1.40–1.68) and patients receiving a NNRTI + NRTI regimen (OR: 1.49, 95% CI: 1.35–1.64).

### Cumulative drug dose trend according to the ART regimen

**Figure 2:**
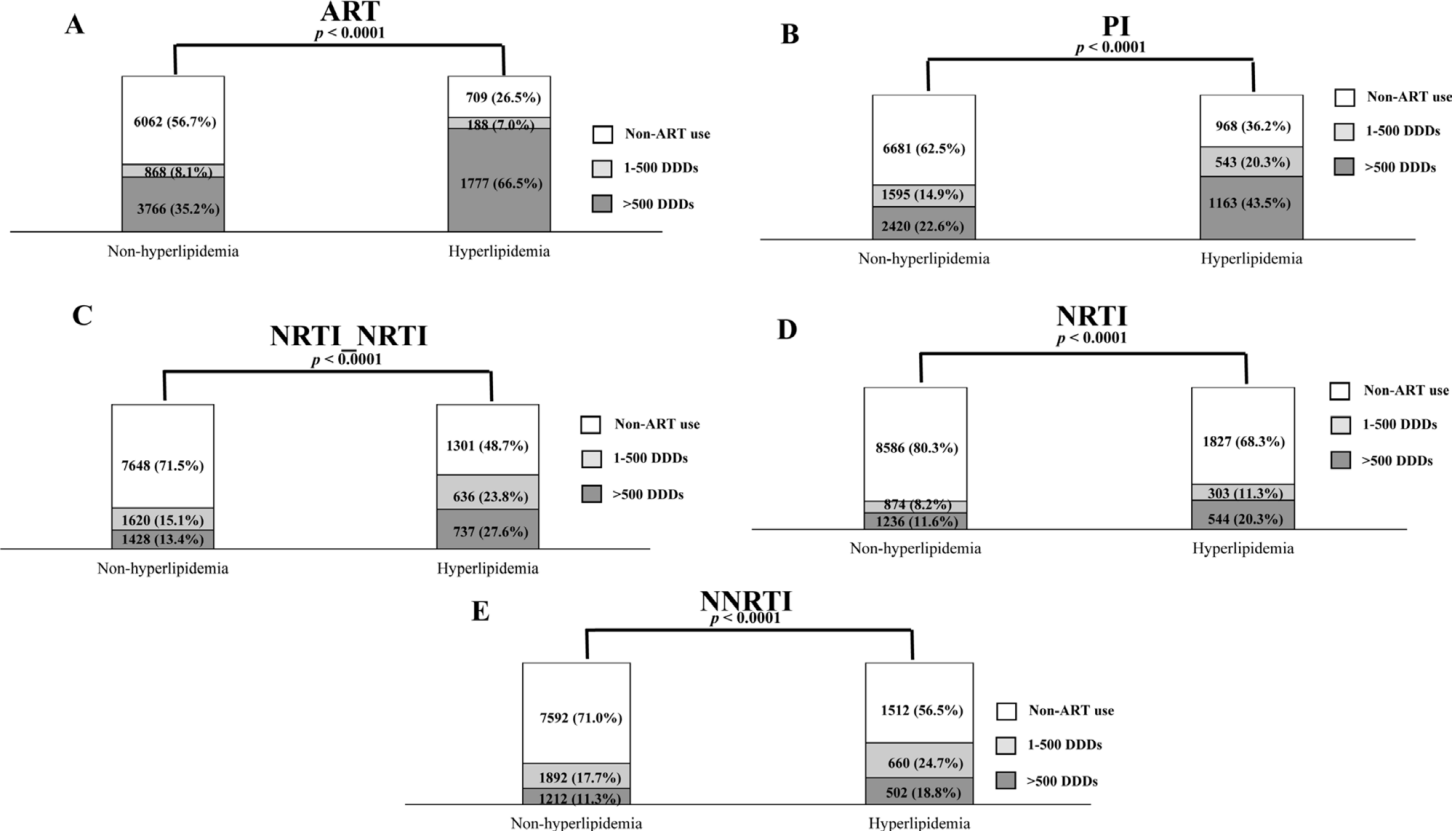
Trends in the distribution of drug cumulative doses between the hyperlipidemia and non-hyperlipidemia groups according to the ART regimens (**A**) including PI (**B**), NRTI/NRTI (**C**), NRTI (**D**), and NNRTI (**E**).

We assessed the cumulative dose distributions between the hyperlipidemia and non-hyperlipidemia groups according to the ART regimen (Figure 2). The percentages of ART use (cumulative dose > 500 DDDs) increased from 35.2% in the non-hyperlipidemia group to 66.5% in the hyperlipidemia group (Figure 2A). The percentages of the PI-only regimen (cumulative dose > 500 DDDs) increased from 22.6% for the non-hyperlipidemia group to 43.5% for those in the hyperlipidemia group (Figure 2B). The percentages of the NRTI/NRTI-containing regimen (cumulative dose > 500 DDDs) increased from 13.4% for the non-hyperlipidemia group to 27.6% for the hyperlipidemia group (Figure 2C). The percentages of the NRTI-containing regimen (cumulative dose > 500 DDDs) increased from 11.6% for the non-hyperlipidemia group to 20.3% for the hyperlipidemia group (Figure 2D). The percentages of the NNRTI-containing regimen (cumulative dose > 500 DDDs) increased from 11.3% for the non-hyperlipidemia group to 18.8% for the hyperlipidemia group (Figure 2E). These results suggest that an increasing cumulative drug dose was observed in regimens containing PI, NRTI/NRTI, NRTI, and NNRTI in the hyperlipidemia group when compared with the non-hyperlipidemia group (Figure 2B–2E).

## DISCUSSION

This is the first, longitudinal, comprehensive, and population-based study to describe the cumulative effect of different types of ART regimens on hyperlipidemia risk in the Taiwanese HIV/ART cohort. Our study found that the matched hyperlipidemia group was associated with a higher number of cases of ART usage and an increased incidence in respiratory disease and diabetes comorbidities. Patients were at a higher hyperlipidemia risk when they had a higher ART dosage, use, and adherence, in a dose-dependent manner. For single ART regimens, patients receiving a NRTI/NRTI-containing regimen had the highest hyperlipidemia risk, followed by PI-containing and NNRTI-containing regimens. For the two-combination ART regimens, patients receiving a NRTI/NRTI + PI regimen had the highest hyperlipidemia risk, followed by NRTI + NRTI and NRTI + PI regimens. In addition, an increasing cumulative drug dose was observed in PI, NRTI/NRTI, NRTI, and NNRTI regimens in the hyperlipidemia group when compared with the non-hyperlipidemia group. Our results suggest that cumulative ART use, ART adherence, and the type of regimen may increase hyperlipidemia risk among HIV-infected patients in the highly active antiretroviral therapy (HAART) era in Taiwan.

Hyperlipidemia has been associated with genetic causes [23], or with other underlying comorbidities including respiratory diseases and diabetes [24–26]. In addition, ART-associated hyperlipidemia can present as TC, high TG, increased plasma LDL-C, increased VLDL, increased apoB, and variable levels of plasma HDL-C [14, 15, 27]. Here, our study paralleled these previous studies, demonstrating that the hyperlipidemia group had a higher level of ART usage. In addition, a meta-analysis of previous studies and our study showed that patients with ART use had a higher hyperlipidemia risk than patients who were not using ART (Supplementary Table 3 and Supplementary Figures 1–2) [28, 29]. Our results also suggest that HIV patients may have a higher hyperlipidemia risk when they receive ART regimens in dose- and adherence- dependent manners, especially in the cases of PI, NRTI, and NNRTI regimens. In addition, it has been found that better ART adherence is associated with a higher risk of subsequent alterations to the adipose tissue [30, 31].

For single ART regimens, patients receiving a NRTI/NRTI- containing regimen had the highest hyperlipidemia risk, followed by PI-containing and NNRTI-containing regimens. NRTI/NRTI regimen treatment (involving two NRTI combined treatment) was associated with the highest risk of hyperlipidemia. NRTI/NRTI includes tenofovir disoproxil/emtricitabine, lamivudine/abacavir, and zidovudine/lamivudine [32]. Our detailed information firstly suggested that among these three NRTI/NRTI-containing regimens, zidovudine/lamivudine and lamivudine/abacavir, were the significant risk factors of hyperlipidemia (Supplementary Tables 1 and 2). Patients taking lamivudine/abacavir containing regimens have been previous characterized with hyper cholesterol and it was suggested that they switch to efavirenz/emtricitabine/tenofovir disoproxil to improve their blood lipid parameters [33].

PI-containing regimen treatment was the second highest risk of hyperlipidemia. PI comprises atazanavir, darunavir, indinavir, lopinavir, nelfinavir, ritonavir, saquinavir, and tipranavir [34]. Patients who receive PI often present with higher levels of triglyceride and LDL-c, lower levels of HDL-c, and this has been associated with peripheral lipodystrophy, hyperlipidemia, and insulin resistance [35–37]. The possible metabolic mechanism is probably due to the fact that PI may inhibit lipogenesis and adipocyte differentiation [37]. In addition, PI appears to bind to the LDL receptor-related protein (LRP), and thus inhibit the function of the LRP-lipoprotein lipase complex (the cleavage of fatty acids from plasma triglycerides) [35]. PI-induced abnormal lipid metabolism may occur due to the alterations of genes in the adipocytes and hepatocytes through sterol regulatory element-binding proteins (SREBPs), cytoplasmic retinoic-acid binding proteins (CRABP-1), peroxisome proliferator activated receptors (PPARs), and apoCIII [35, 38, 39]. Our detailed information firstly showed that among these eight PI-containing regimens, lopinavir/ritonavir was associated with the highest risk of hyperlipidemia, followed by ritonavir (Supplementary Tables 1 and 2). Our results are in agreement with previous studies that identified an increased risk of hyperlipidemia associated with HIV-infected patients on lopinavir/ritonavir monotherapy [38]. Lopinavir/ritonavir has been reported to induce a reduction in peripheral adipose depots in mice via increasing SREBP-1c protein expression [39, 40] and to impair physical strength in association with reduced IGF1 expression in the skeletal muscle of older mice [41]. Our findings also suggested that ritonavir was the second highest risk of hyperlipidemia (Supplementary Tables 1 and 2). Ritonavir has also been associated with hyperlipidemia and premature atherosclerosis [42, 43]. Ritonavir was shown to impair adipocyte differentiation *in vitro* by increasing the level of active mature ADD-1/SREBP-1 [44].

NNRTI contains efavirenz, etravirine, and nevirapine [45]. Our detailed information firstly showed that among these three NNRTI-containing regimens, efavirenz was associated with the highest risk of hyperlipidemia (Supplementary Tables 1 and 2). Patients prescribed efavirenz have been found to show mild dyslipidemic effects, with increases in total cholesterol, LDL-C, and HDL-C [46, 47]. Efavirenz also impairs adipocyte differentiation *in vitro* [48]. Preadipocytes treated with efavirenz fail to accumulate cytoplasmic triacylglycerol droplets via suppression of SREBP-1c protein expression, which contributes to adipose tissue atrophy.

NRTI-containing regimen treatment was the fourth highest risk of hyperlipidemia. NRTI contains abacavir, didanosine, lamivudine, stavudine, zalcitabine, zidovudine, and tenofovir disoproxil. NRTI regimens are also associated with alterations in hypertriglyceridemia and body fat deposition [49, 50]. Our study suggested that five of these seven NRTI-containing regimens, abacavir, didanosine, lamivudine, stavudine, and zidovudine, were associated with the risk of hyperlipidemia (Supplementary Tables 1 and 2). Among them, didanosine has been previously found to induce hyperlipidemia [51–53]. Stavudine-containing regimens have also been previously shown to be associated with hyperlipidemia, through a mechanism involving mitochondrial respiratory chain dysfunction and reduced SREBPs and adiponectin levels [54]. Similarly, lamivudine-containing regimens induced higher levels of cholesterol [33].

For combination ART regimens, patients receiving a NRTI/NRTI + PI regimen had the highest hyperlipidemia risk. In addition, a meta-analysis of previous studies and our study identified that patients on NRTI/NRTI + PI regimens had a higher hyperlipidemia risk than patients who did not have NRTI/NRTI + PI regimens (Supplementary Table 3 and Supplementary Figures 3–4). Our detailed information firstly showed that among these 15 NRTI/NRTI + PI regimens, lopinavir/ ritonavir + zidovudine/ lamivudine was associated with the highest risk of hyperlipidemia, followed by lamivudine/ abacavir + lopinavir/ ritonavir (Supplementary Tables 1 and 2). Further functional characterization of these two combined ART regimens in pre-adipocyte differentiation is required. To our knowledge, this is the first, longitudinal and comprehensive study to describe the effect of different types of ART regimens on hyperlipidemia risk by using a database that included all HIV infected patients in Taiwan. Moreover, our results suggest that patients who received a NRTI/NRTI + PI regimen exhibited the highest hyperlipidemia risk.

Our results provide a comprehensive assessment of hyperlipidemia risk associated with ART regimens in Taiwanese patients and may provide future directions regarding alternative ART regimens to avoid the additional risk of hyperlipidemia. Furthermore, lipid-lowering agents such as statin, fibrate, complementary herbal medicine, and lipid-based nutrient supplements to reduce the additional risk of hyperlipidemia are increasingly acknowledged for HIV infected patients to improve survival as well as hyperlipidemia risk [55, 56]. The efficacy and safety of statins in HIV infected patients with hyperlipidemia has been investigated and these are suggested for use in children, adolescents and young adults [57].

By using the National Health Insurance Research Database in Taiwan, we were able to investigate the hyperlipidemia risk associated with cumulative ART usage, adherence, and their combination. Limitations of this study included a lack of blood physiological and biochemical measures in this database, such as TC, TG, LDL-C, VLDL, apoB, and variable levels of plasma HDL-C. Other limitations included the lack of information regarding the inflammatory state and immune activation of these HIV infected patients, genetic and environmental factors (including levels of job stress and exercise), personal histories (including education and body mass index), and potential disease misclassifications [58–62]. In this study, we used a population-based database to investigate the cumulative effect of ART and the effect of ART regimens on hyperlipidemia risk in a Taiwanese HIV/ART cohort. Our study provides evidence that cumulative ART use, adherence to ART, and the type of regimen increases hyperlipidemia risk in HIV-infected patients in a dose and adherence-dependent manners in the Taiwanese HAART era.

## MATERIALS AND METHODS

### Study population

This study was designed as a population-based retrospective cohort and nested case-control study, and its purpose was to explore the cumulative effect of ART regimens and ART adherence on hyperlipidemia risk in HIV-infected patients in Taiwan. HIV-infected patients (*N* = 25,010) were selected from the National Health Insurance (NHI) system in Taiwan (Figure 1). This Taiwanese NHI system provides a database- the National Health Insurance Research database (NHIRD; http://nhird.nhri.org.tw/), which has been established since 1995. This database covers insurance for the majority of the Taiwanese population, and provides valuable information such as age, gender, comorbidities, and prescription patterns etc., for research purposes to scientists.

Information on 25,010 HIV-infected patients (ICD-9-CM: 042-044, 079, and V08 code) was requested from the NHI, Taiwan. There were 22,755 HIV-infected Taiwanese patients between 1998 and 2011. Individuals with hyperlipidemia prior to HIV infection (*N* = 726) were excluded. After application of these criteria, 22,029 participants were finally included in the study cohort. The date of satisfying the diagnosis of HIV infection was designated as the index date. The cases were defined as patients who had first reported hyperlipidemia between 1998 and 2011, after HIV infection (*N* = 2,706; Figure 1). Hyperlipidemia was defined according to the ICD-9-CM: 272. The controls were HIV-infected patients with no record of hyperlipidemia (*N* = 19,323). With the aim of having four controls per case patient, the incidence-density sampling match method was applied to match the hyperlipidemia and non-hyperlipidemia groups. After matching these two groups for age, gender, and the first diagnosis date of HIV infection, 2,674 hyperlipidemia and 10,696 non-hyperlipidemia patients were included in this study. This study was approved for the purchase and investigation of the National Health Insurance Research Database (NHIRD) by the Human Studies Committee of China Medical University Hospital, Taichung, Taiwan. No informed consent was given *because the data were analyzed anonymously*.

### Data collection

The demographic data collected included age, gender, follow-up years, ART usage, and comorbidities. Comorbidities present in the patients prior to their subsequent HIV diagnosis were defined as follows: cardio-cerebrovascular disease (ICD-9-CM: 410, 412, 428, 441, 443.9, 430-438, 785.4, V43.4, and 38.48 (P)), respiratory diseases (ICD-9-CM: 490–496, 500–505, and 506.4), rheumatic diseases (ICD-9-CM: 710.0, 710.1, 710.4, 714.0–714.2, 714.81, and 725), digestive diseases (ICD-9-CM: 531–534), diabetes (ICD-9-CM: 250.0–250.3, and 250.7), renal disease (ICD-9-CM: 582, 583–583.7, 585, 586, and 588), liver diseases (ICD-9-CM: 571.2, 571.4–571.6, 070.4, 070.5, and 070.7), and cancer (ICD-9-CM: 140–172, 174–195.8, and 200-208) (Table 1).

### Statistical analysis

The demographic data were expressed as counts and percentages for categorical variables, for both the hyperlipidemia and non-hyperlipidemia groups. These included age, gender, ART usage, and comorbidities, which were analyzed by chi-squared tests. The follow-up years for both the groups were expressed as continuous variables and analyzed by the student *t*-test. The distribution of the cumulative drug dose trend between the hyperlipidemia and non-hyperlipidemia groups was analyzed by chi-squared tests. Conditional logistic regression analysis was applied to explore the effect of cumulative ART dose and adherence to ART on hyperlipidemia risk in HIV-infected patients. Conditional logistic regression analysis was also applied to explore the effect of different types of ART regimens on hyperlipidemia risk in HIV-infected patients. In addition, conditional logistic regression analysis was applied to explore the effect of different combination ART regimens on hyperlipidemia risk in HIV-infected patients. All *p*-values < 0.05 were considered significant. All data management and statistical analyses were performed using the Statistical Analysis System (SAS) software (version 9.3; SAS Institute, Cary, NC, USA).

## SUPPLEMENTARY MATERIALS FIGURES AND TABLES





## Abbreviations

HIV: human immunodeficiency virus
AIDS: acquired immune deficiency syndrome
ART: antiretroviral therapy
NRTI: Nucleoside reverse transcriptase inhibitors
NNRTI: Non-Nucleoside Reverse Transcriptase Inhibitors
PI: Protease inhibitor
CI: confidence interval
