# Supplementary material for: Effect of antiretroviral therapy use and adherence on the risk of hyperlipidemia among HIV-infected patients, in the highly active antiretroviral therapy era
Source: Oncotarget. 2017 Nov 15;8(63):106369–81. doi: 10.18632/oncotarget.22465 (PMC5739740; doi:10.18632/oncotarget.22465)
Supplement: Supplementary file 1 [file oncotarget-08-106369-s001.pdf]

Effect of antiretroviral therapy use and adherence on the risk of hyperlipidemia among HIV-infected patients, in the highly active antiretroviral therapy era

SUPPLEMENTARY MATERIALS

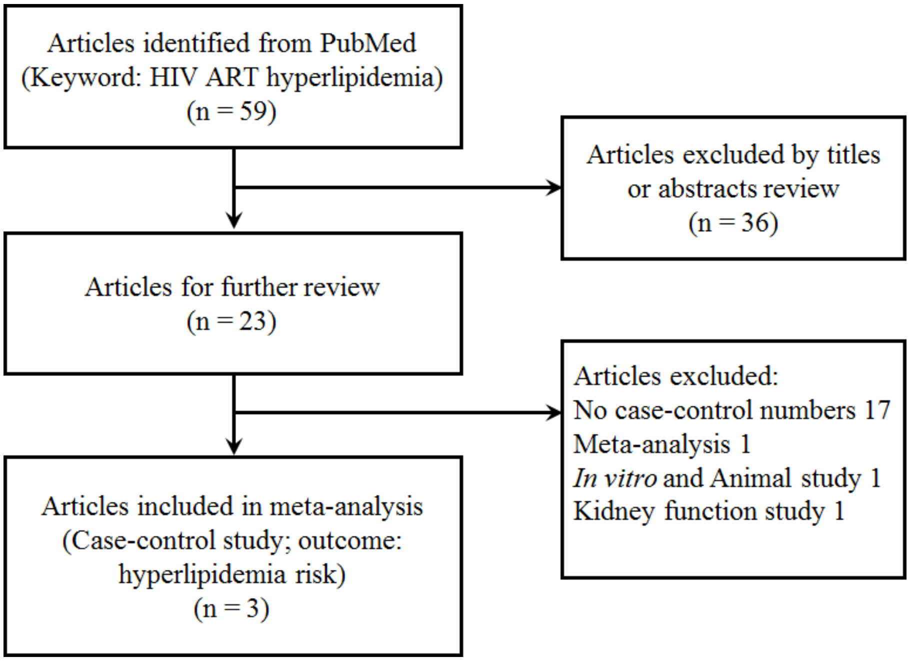

Supplementary Figure 1: Flow chart of the selection process for the meta-analysis in this study.

A

Model	Study	ART use		Non-ART use		Odds ratio	Lower limit	Upper limit	*p*-Value
		Hyperlipidemia	Non-hyperlipidemia	Hyperlipidemia	Non-hyperlipidemia				
	Mendicino CC et al., 2016	13	15	9	19	1.830	0.619	5.411	0.275
	Carey RA et al., 2013	24	26	10	48	**4.430**	**1.840**	**10.668**	***0.001***
**Fixed**						**3.120**	**1.576**	**6.175**	***0.001***

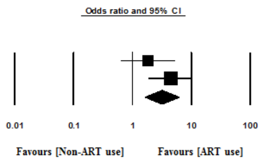

Test for heterogeneity: Q-value 1.542; *P*-value: 0.214

B

Model	Study	ART use		Non-ART use		Odds ratio	Lower limit	Upper limit	*p*-Value
		Hyperlipidemia	Non-hyperlipidemia	Hyperlipidemia	Non-hyperlipidemia				
	Mendicino CC et al., 2016	13	15	9	19	1.830	0.619	5.411	0.275
	Carey RA et al., 2013	24	26	10	48	**4.430**	**1.840**	**10.668**	***0.001***
	Our study	1965	709	4634	6062	**3.630**	**3.305**	**3.986**	***<0.0001***
**Fixed**						**3.620**	**3.299**	**3.972**	***<0.0001***

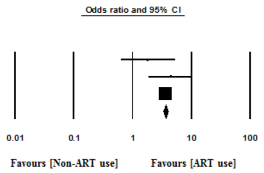

Test for heterogeneity: Q-value 1.747; *P*-value: 0.422

Supplementary Figure 2: Forest plots of ART use versus non-ART use (**A** and **B**).

A

Model	Study	NRTI/NRTI + PI		Non-(NRTI/NRTI + PI)		Odds ratio	Lower limit	Upper limit	*p*-Value
		Hyperlipidemia	Non-hyperlipidemia	Hyperlipidemia	Non-hyperlipidemia				
	Mendicino CC et al., 2016	8	14	14	20	0.820	0.272	2.475	0.725
	Jones R et al., 2005	60	366	111	1127	**1.660**	**1.186**	**2.323**	***0.003***
**Fixed**						**1.564**	**1.134**	**2.157**	***0.006***

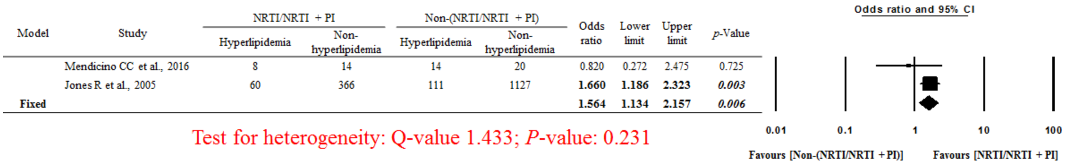

Test for heterogeneity: Q-value 1.433; *P*-value: 0.231

B

Model	Study	NRTI/NRTI + PI		Non-(NRTI/NRTI + PI)		Odds ratio	Lower limit	Upper limit	*p*-Value
		Hyperlipidemia	Non-hyperlipidemia	Hyperlipidemia	Non-hyperlipidemia				
	Mendicino CC et al., 2016	8	14	14	20	0.820	0.272	2.475	0.725
	Jones R et al., 2005	60	366	111	1127	**1.660**	**1.186**	**2.323**	***0.003***
	Our study	1283	2990	1391	7706	**2.380**	**2.184**	**2.594**	***<0.0001***
**Random**						**1.854**	**1.242**	**2.766**	***0.003***

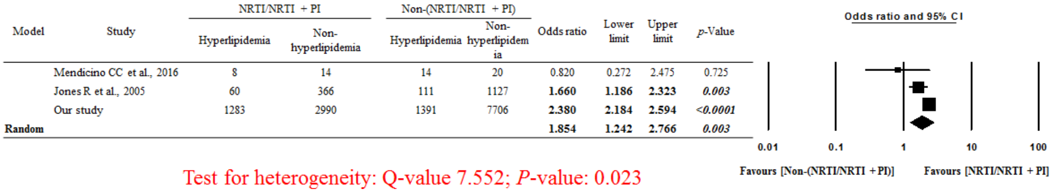

Test for heterogeneity: Q-value 7.552; *P*-value: 0.023

Supplementary Figure 3: Forest plots of NRTI/NRTI + PI versus non-(NRTI/NRTI + PI) (**A** and **B**).

A

Model	Study	NRTI/NRTI + NNRTI		Non-(NRTI/NRTI + NNRTI)		Odds ratio	Lower limit	Upper limit	*p*-Value
		Hyperlipidemia	Non-hyperlipidemia	Hyperlipidemia	Non-hyperlipidemia				
	Mendicino CC et al., 2016	14	20	8	14	1.230	0.409	3.695	0.712
	Jones R et al., 2005	84	754	87	739	0.950	0.692	1.304	0.751
**Fixed**						0.969	0.715	1.314	0.839

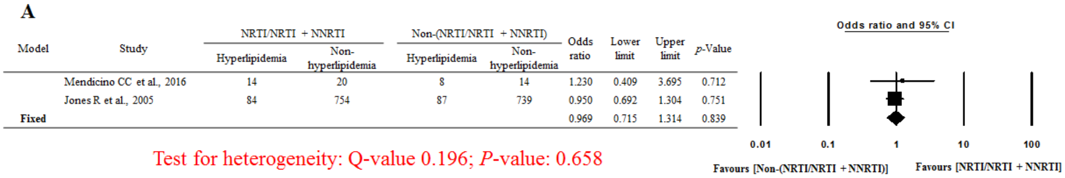

Test for heterogeneity: Q-value 0.196; *P*-value: 0.658

B

Model	Study	NRTI/NRTI + NNRTI		Non-(NRTI/NRTI + NNRTI)		Odds ratio	Lower limit	Upper limit	*p*-Value
		Hyperlipidemia	Non-hyperlipidemia	Hyperlipidemia	Non-hyperlipidemia				
	Mendicino CC et al., 2016	14	20	8	14	1.230	0.409	3.695	0.712
	Jones R et al., 2005	84	754	87	739	0.950	0.692	1.304	0.751
	Our study	931	2768	1743	7928	1.53	1.401	1.671	***<0.0001***
**Random**						1.241	0.833	1.851	0.288

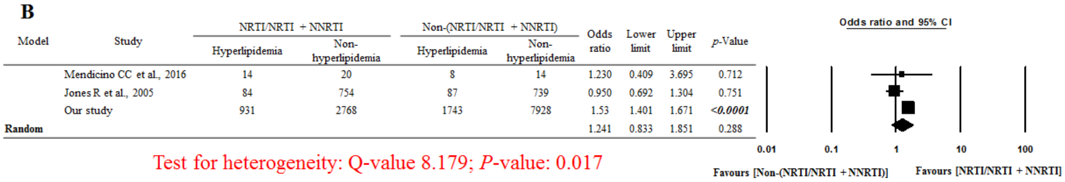

Test for heterogeneity: Q-value 8.179; *P*-value: 0.017

Supplementary Figure 4: Forest plots of NRTI/NRTI + NNRTI versus non-(NRTI/NRTI + NNRTI (**A** and **B**).

Supplementary Table 1: Hyperlipidemia risk in HIV-infected patients according to single type of detail ART drug name

ART regimen	Detail ART drug name	Hyperlipidemia *N* = 2,674		Non- hyperlipidemia N = 10,696		OR	95% CI	*p*- value
		N	%	*N*	%			
**Non-ART use**		709	26.5%	6062	56.7%			
**NNRTI**								
	**efavirenz**	962	36.0%	2503	23.4%	1.87	(1.71, 2.06)	***<0.0001***
	etravirine	ND	ND	ND	ND	ND	ND	ND
	**nevirapine**	333	12.5%	1019	9.5%	1.35	(1.19, 1.55)	***<0.0001***
**NRTI**								
	**abacavir**	214	8.0%	557	5.2%	1.57	(1.33, 1.85)	***<0.0001***
	**didanosine**	358	13.4%	851	8.0%	1.81	(1.59, 2.07)	***<0.0001***
	**lamivudine**	767	28.7%	1886	17.6%	1.9	(1.72, 2.1)	***<0.0001***
	**stavudine**	476	17.8%	1162	10.9%	1.79	(1.6, 2.02)	***<0.0001***
	zalcitabine	32	1.2%	102	1.0%	1.28	(0.86, 1.9)	0.2331
	**zidovudine**	162	6.1%	446	4.2%	1.5	(1.24, 1.8)	***<0.0001***
	tenofovir disoproxil	36	1.3%	107	1.0%	1.35	(0.92, 1.98)	0.1212
**NRTI/NRTI**								
	tenofovir disoproxil/ emtricitabine	1	0.0%	ND	ND	ND	ND	ND
	**lamivudine/abacavir**	638	23.9%	1306	12.2%	2.27	(2.04, 2.53)	***<0.0001***
	**zidovudine/ lamivudine**	1316	49.2%	3243	30.3%	2.32	(2.12, 2.54)	***<0.0001***
**PI**								
	**atazanavir**	308	11.5%	801	7.5%	1.58	(1.37, 1.82)	***<0.0001***
	darunavir	7	0.3%	12	0.1%	2.35	(0.92, 5.97)	0.0738
	**indinavir**	258	9.6%	735	6.9%	1.46	(1.26, 1.7)	***<0.0001***
	**lopinavir/ ritonavir**	974	36.4%	1756	16.4%	3.01	(2.73, 3.31)	***<0.0001***
	**nelfinavir**	156	5.8%	385	3.6%	1.65	(1.36, 2)	***<0.0001***
	**ritonavir**	308	11.5%	748	7.0%	1.74	(1.51, 2.01)	***<0.0001***
	**saquinavir**	151	5.6%	441	4.1%	1.42	(1.17, 1.72)	0.0003
	tipranavir	1	0.0%	1	0.0%	4.11	(0.26, 65.72)	0.3178
**Other ART**								
	raltegravir	6	0.2%	12	0.1%	2.05	(0.77, 5.48)	0.1511
	enfuvirtide	1	0.0%	3	0.0%	1.35	(0.14, 12.96)	0.7963

N, number; ART, antiretroviral therapy; OR, odds ratio; CI, confidence interval; NNRTI, non-nucleoside reverse transcriptase inhibitors; NRTI, nucleoside/nucleotide reverse transcriptase inhibitors; PI, protease inhibitors; ND, not determined.

Adjusted for age, gender, Charlson's comorbidity.

Significant *p*-values ($p < 0.0001$) are highlighted in bold italic font.

NNRTI includes efavirenz, etravirine, and nevirapine; NRTI includes abacavir, didanosine, lamivudine, stavudine, zalcitabine, zidovudine, and tenofovir disoproxil; NRTI/NRTI includes tenofovir disoproxil/emtricitabine, lamivudine/abacavir, and zidovudine/lamivudine; PI includes atazanavir, darunavir, indinavir, lopinavir/ ritonavir, nelfinavir, ritonavir, saquinavir, and tipranavir; other ART includes raltegravir and enfuvirtide.

Supplementary Table 2: hyperlipidemia risk in HIV-infected patients according to combination of 2 ART regimens (detail ART drug name). See Supplementary_Table_2

Supplementary Table 3: Characteristics of included studies

Studies	Mendicino CC *et al*., 2016	Jones R *et al*., 2005	Carey RA *et al*., 2013	Our study
**Type of study**	Retrospective cohort with case-control study	Longitudinal, prospective cohort	cross-sectional study	Nested, retrospective cohort with case-control study
**Age range**	≥18 years	39.2 years	33–39 years	37–39 years
**Countries included**	Brasil	UK	India	Taiwan
**Period of enrollment**	2012 to 2013	1996–2003	2011–2013	2000–2010
**Sample size**	100	1,664	108	13,370
**Intervention**	ART use, NRTI/NRTI + PI, NRTI/NRTI + NNRTI	NRTI/NRTI + PI, NRTI/NRTI + NNRTI	ART use	ART use, NRTI/NRTI + PI, NRTI/NRTI + NNRTI
**Comparators**	Non-ART use, non-NRTI/NRTI + PI, non-NRTI/NRTI + NNRTI	non-NRTI/NRTI + PI, non-NRTI/NRTI + NNRTI	Non-ART use	Non-ART use, non-NRTI/NRTI + PI, non-NRTI/NRTI + NNRTI
**Length of follow-up**	Up to 60 months	Up to 5.75 years	Up to 31 months	Up to 10 years
